# LPWAN Cyber Security Risk Analysis: Building a Secure IQRF Solution

**DOI:** 10.3390/s23042078

**Published:** 2023-02-12

**Authors:** Mohammed Bouzidi, Ahmed Amro, Yaser Dalveren, Faouzi Alaya Cheikh, Mohammad Derawi

**Affiliations:** 1 Department of Electronic Systems, Norwegian University of Science and Technology, 2821 Gjøvik, Norway; 2 Department of Information Security and Communication Technology, Norwegian University of Science and Technology, 2821 Gjøvik, Norway; 3 Department of Electrical and Electronics Engineering, Atilim University, Incek Golbasi, 06830 Ankara, Turkey; 4 Department of Computer Science, Norwegian University of Science and Technology, 2821 Gjøvik, Norway

**Keywords:** LPWAN, Internet of Things (IoT), IQRF, FMEA, security

## Abstract

Low-power wide area network (LPWAN) technologies such as IQRF are becoming increasingly popular for a variety of Internet of Things (IoT) applications, including smart cities, industrial control, and home automation. However, LPWANs are vulnerable to cyber attacks that can disrupt the normal operation of the network or compromise sensitive information. Therefore, analyzing cybersecurity risks before deploying an LPWAN is essential, as it helps identify potential vulnerabilities and threats as well as allowing for proactive measures to be taken to secure the network and protect against potential attacks. In this paper, a security risk analysis of IQRF technology is conducted utilizing the failure mode effects analysis (FMEA) method. The results of this study indicate that the highest risk corresponds to four failure modes, namely compromised end nodes, a compromised coordinator, a compromised gateway and a compromised communication between nodes. Moreover, through this methodology, a qualitative risk evaluation is performed to identify potential security threats in the IQRF network and propose countermeasures to mitigate the risk of cyber attacks on IQRF networks.

## 1. Introduction

The Internet of Things (IoT) is a communication paradigm that refers to a network of many interconnected electronic devices that are each equipped with microprocessors, sensors, and data storage units. However, due to the heterogeneous network environment, it is necessary to ensure the interconnection between the devices [1]. To this end, an aggregated traffic modeling approach [2] was conceptualized that enables a large number of devices to share data through a gateway operated by a wireless technology for short- or long-range data transmission. For short-range data transmission, basic communication protocols such as Bluetooth, Wi-Fi, or ZigBee are primarily used. For long-range data transmission on the other hand, mobile network technologies can be used at the expense of a high resource overhead. Therefore, long range wireless technologies such as low-power wide area networks (LPWAN) have been developed to fill the gap between short-range communication protocols and mobile networks technologies [3].

The past few years have seen a widespread diffusion of novel LPWAN technologies. Some LPWAN products, such as LoRa, Sigfox, On-Ramp Wireless, Weightless, and many others [4,5], have been gaining momentum and commercial interest as enabling technologies for the IoT. Alternatively, some other emerging technologies of LPWAN are gaining maturity and waiting to be investigated. Intelligent connectivity using radio frequency (IQRF) is one of these technologies [6,7]. IQRF is a recently developed IoT platform for wireless connectivity operating in sub-GHz ISM bands. It provides a full mesh network, terminating into gateways which in turn upload data to the cloud through LTE, GSM, or Wi-Fi protocols. This technology could be considered not only as a low-power metropolitan area network (LPMAN), but also as a LPWAN, since it offers cost-effective solutions for medium/long range IoT applications that extend from a few to tens of kilometers [8,9,10].

Although LPWAN technologies provide several benefits, security is still one of the hot topics that needs to be addressed. This is because LPWANs are commonly formed as wireless sensor networks (WSNs), in which the end nodes (IoT devices) will inevitably be the targets of various cyber threats [11,12,13,14]. Security issues arise due to the threats of weaknesses and vulnerabilities being exploited. Therefore, conducting security risk analysis and assessment could be useful prior to the deployment of IoT networks [15].

On the other hand, barring a few works [8,16], IQRF technology has not been scrutinized regarding security aspects for IoT applications. In fact, IQRF security features and the threats that could pose security vulnerabilities in IQRF networks have not yet been studied. In this study, specific enhancements in building a secure IQRF solution are proposed to address the security threats. To this end, security risk analysis for IQRF networks is conducted using the failure mode effects analysis (FMEA) method that is based on the standard IEC 60812:2018 [17]. In this way, IQRF system components that need to be improved are highlighted. In fact, this is the first study that provides useful information based on the fundamental perspectives and failures producing vulnerabilities in IQRF systems. The main contributions of this study are two-fold:

(a) The FMEA method is used to conduct security risk analysis for IQRF networks.
(b) Security risks in IQRF technology are identified, and further actions that can be used to protect IQRF networks from cyber attacks are addressed.

The paper is organized as follows. In Section 2, related works are briefly discussed. The FMEA approach and the experiment setup are introduced in Section 3. Then, the IoT security risk analysis conducted in this study is described in Section 4, where the considered failure modes, existing controls in IQRF technology, expected consequences of failures, possible causes of failures, and suggested solutions are presented. Section 5 discusses the main findings of this study. Finally, Section 6 provides concluding remarks and suggestions for the future work.

## 2. Related Work

The security of IQRF technology has been studied by a very limited number of studies [8,16]. In [8], an in-depth analysis together with experimental measurements and simulations that are related to propagation aspects for indoor and outdoor environments are provided. In addition, the security mechanisms implemented in an IQRF network are briefly described. In [16], the security aspects of IQRF and other well-known IoT technologies and standards are surveyed. The security mechanisms implemented in IQRF are briefly addressed and some of the attacks that could be a potential threat for an IQRF network are outlined. However, there are no studies in the literature that have identified the cybersecurity risks in IQRF technology. To do this, it is necessary to choose a risk analysis approach that is applicable to IoT systems.

Applying risk assessment approaches helps to prioritize and to effectively address the risks within a system. Although there are several steps forming the fundamental process for most of cybersecurity risk assessment approaches, there is still a degree of flexibility in how these steps are implemented. Thus, this has resulted in the emergence of various traditional approaches for this purpose such as fault tree analysis [18], event tree analysis [19], decision matrix risk assessment [20], weighted risk analysis [21], fuzzy set theory [22], Dempster–Shafer theory [23], game theoretic computing [24], cyber security game [25], operationally critical threat asset and vulnerability evaluation (OCTAVE) [26], and FMEA [27]. Before the development of the IoT and WSNs, the use of these approaches was mostly preferred to manage security risks. However, where the complexity, dynamism, and pervasiveness requirements of IoT and WSNs are concerned, there might be some important doubts regarding the use of these approaches, particularly in cyber risk analysis for IoT systems. In this context, the existing popular cyber risk assessment approaches and their suitability towards IoT systems are discussed in [28]. In this work, risks vectors that are related to the IoT are also presented. In addition, a classification method to classify and quantify IoT risks is introduced, with the aim of initiating a risk assessment approach exclusively for IoT systems. Moreover, traditional risks assessment approaches that are suitable for IoT systems are presented by discussing their IoT employment considerations. It was concluded that the FMEA method could be a reasonable choice for cyber risk analysis for IoT systems, among others, due to its easier applicability and usability. Therefore, in this study, the FMEA method is used to conduct security risk analysis for IQRF networks.

The application of the FMEA method to IoT-based systems is scrutinized by a few works in the literature [29,30,31]. In [29], the application of the FMEA method in a honeypot-based cybersecurity experiment in an IoT environment is discussed. The factors that affect the outcome and contribute to the failures of the experiment are identified. The causes of failures, along with their effects, and the possible ways of their mitigation are also discussed. In [30], an FMEA method based on the fuzzy set theory and the gray relational theory is proposed to assess the information security risk of smart city. Similarly, a FMEA-based approach used for security risk assessment on two case studies related to smart buildings is presented in [31]. Overall, the presented work and relevant works are summarized in Table 1.

## 3. The Used Approach and Experiment Design

### 3.1. FMEA/FMECA Approach

FMEA is qualitative analysis technique used to define, identify, and eliminate known or potential threats for cyber systems. It is a proven and widely adopted approach for failure analysis and risk assessment. Moreover, FMEA is a systemic methodology intended to perform mainly three analyzing steps such as identifying and recognizing potential failures by taking in consideration the causes and the effects involved in the failures, evaluating the prioritized identified failure modes, since failures do not have the same degree, and identifying and suggesting actions that can reduce or eliminate failures from occurring [32]. Commonly, the basic steps of FMEA are as follows:

- Failure modes identify all possible failure modes of a cyber system.
- Failure effects identify resulting effects of the potential failures.
- Failure causes identify the possible causes for the failure modes.
- Employed controls identify the ways to detect, mitigate, or prevent failures.
- Risk evaluations assess the risk level associated with each of the failure modes based on a set of established criteria.

On the other hand, failure mode effects and criticality analysis (FMECA) provides an additional analysis step over FMEA, focusing on the criticality of failure modes [33]. While FMEA is a qualitative analysis approach, FMECA includes quantitative values, adding an evaluation part that aids in taking coherent operational decisions [34]. As an extension to FMEA, the FMECA approach enables quantifying the entity of a specific defect in the system using numerical indices. These indices are:

- Severity (Sev), encompassing the consequence of the failure when it happens in cyber systems. A low number corresponds to a low impact, while a high number corresponds to a high impact.
- Occurrence (Occ), defining the probability or frequency of a failure occurring during the expected lifetime of the cyber system. A low number is not likely to occur, while a high number is inevitable.
- Detection (Det), defining the probability of the failure being detected and acted upon before it happens. A low number is very likely to be detected, while a high number is not likely to be detected.

The three indices, ranging from 1 to 10 each, are then combined together in one quantity called the risk priority number (RPN), which reflects the priority of the identified failure modes [35]. The RPN is calculated by multiplying the three indices as given in the following:

(1) RPN=Sev×Occ×Det.

**Figure 1:**
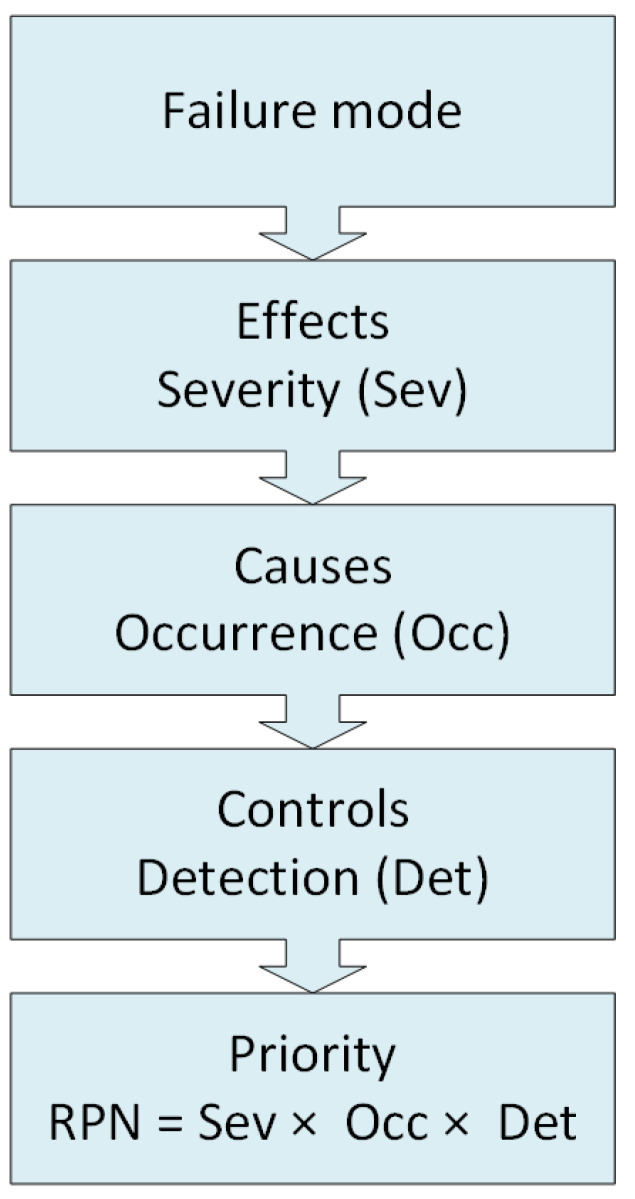
Important FMEA/FMECA tasks [17,32].

In Figure 1, important FMEA tasks and RPN evaluation criteria are summarized.

It is worth mentioning that FMECA is not addressed in this paper, since the criticality analysis requires a specific use case description to estimate the criticality parameters within the context of the use case. Therefore, in this paper, the analysis is limited to using FMEA as defined by the IEC 60812:2018 standard [17] for guiding the process and qualitatively analyzing the cyber risks for IQRF technology.

### 3.2. The Experiment Design

**Figure 2:**
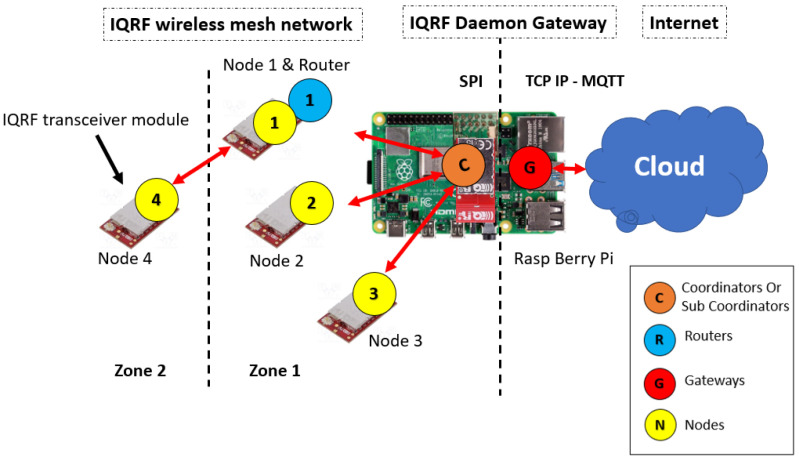
IQRF system architecture [6].

**Figure 3:**
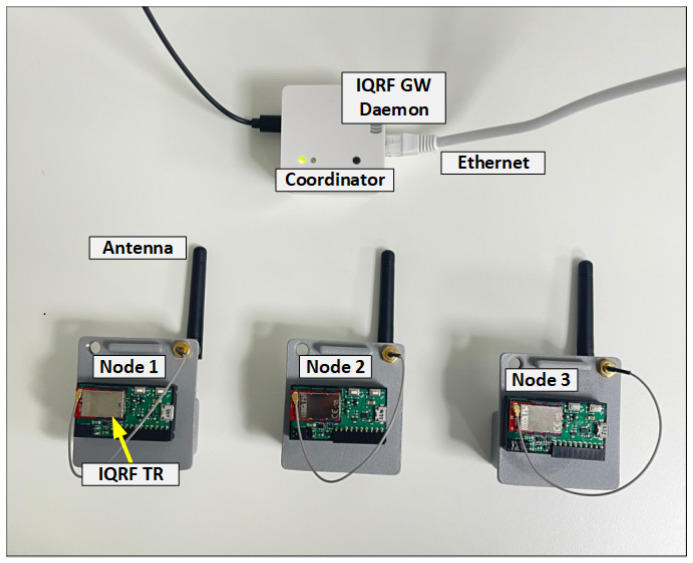
The experiment setup. Three IQRF nodes, one coordinator node, and one gateway.

The network architecture of an IQRF solution is made up of three levels, namely (i) an IQRF transceiver (TR) module including end nodes and the coordinator node, (ii) an IQRF gateway software, and (iii) cloud systems, as shown in Figure 2. More details about each level are provided in Section 4.1. To implement the FMEA approach, the setup is built by following the structure in Figure 2. In this setup, three nodes (TRs) are configured as end nodes using basic configurations such as the transmission power, the communication channel, access passwords, and other basic parameters in building an IQRF network (https://doc.iqrf.org/IQMESH-Network-deployment/, accessed on 26 January 2023). A fourth node (TR) is configured as a coordinator. Similarly, the same basic configuration of end nodes are used for the coordinator node. In addition, a special plug-in that implements the principal functionalities of the coordinator, such as network communications and network management, is uploaded to this node. End nodes are then linked to the coordinator node using the web interface, called Webapp, that is installed on the gateway. The coordinator node is mounted on the gateway as the IQRF network architecture requires. Figure 3 shows the experiment setup on which the analysis is performed.

## 4. Security Analysis of IQRF Technology

**Figure 4:**
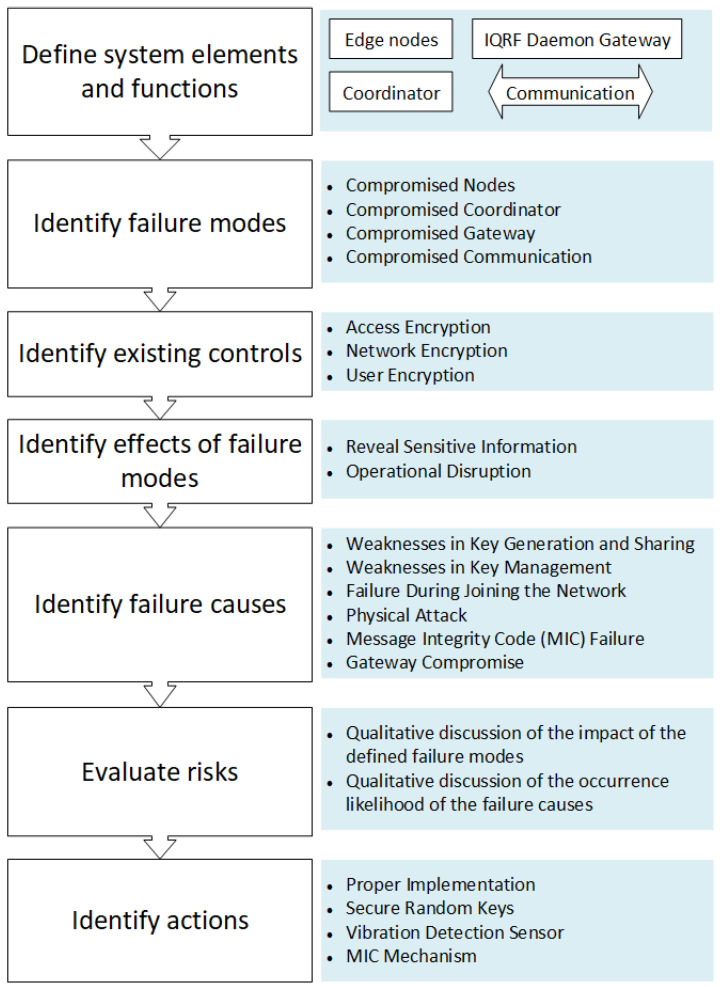
Steps in the FMEA process including the summarized results.

In this section, the method and the findings of the conducted security analysis for the IQRF technology are presented. It is worth mentioning that the cloud system security analysis is not part of this study. Only the cyber risks of IQRF WSN, the communications between the nodes, and a part of the gateway are analyzed. The analysis process is depicted in Figure 4, with brief details about the followed steps in this work. Notably, FMEA focuses on failures in a system during the cybersecurity analysis, and attacks are the general term of undesired behavior (i.e., failures). In this paper, the terms failures and attacks are used interchangeably.

### 4.1. Define System Elements and Functions

In order to apply the FMEA method, the system shown in Figure 2 is divided into subsystems by identifying each of system elements mentioned in the experimental design section.

**Figure 5:**
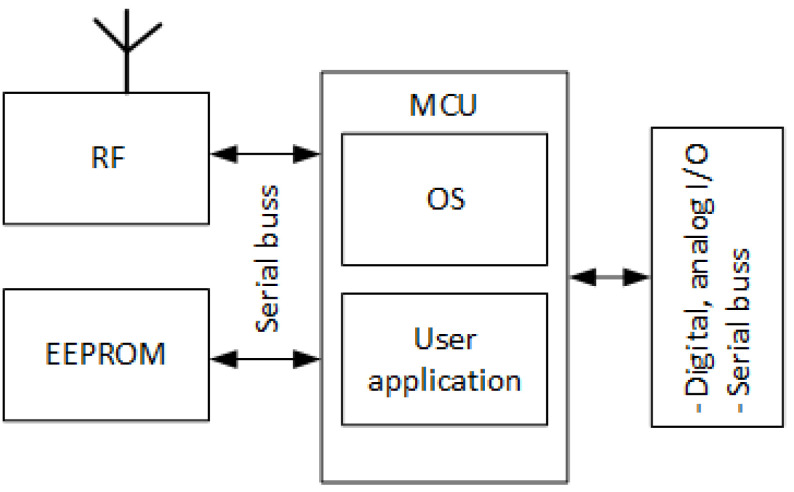
IQRF transceiver module architecture https://www.iqrfalliance.org/technology, (accessed on 4 November 2022).

The IQRF TR module represents the end nodes in an IQRF WSN network. It also plays the role of a coordinator that manages the end node communications in the network, such as joining or leaving the network and other networking procedures. However, the coordinator configuration is different than that of the end nodes. IQRF TRs operate in the 868MHz license-free ISM band using GFSK modulation. Its highly integrated, ready-to-use design contains mainly a micro controller unit (MCU), an RF circuitry, and optional components. Each TR module (node) in the network has a built-in operating system (OS) installed in the MCU for the RF (wireless) communication system management and complex mesh routing protocol called IQMESH [36]. The routing protocol is based on an optimized directional flooding and its main advantages are higher throughput and much higher robustness, which primarily becomes apparent in industrial and other applications where a high reliability is strictly required. In some cases, IQRF nodes are used to measure physical quantities when different sensors are mounted on the IQRF TR or to control remotely external systems. Moreover, IQRF TR modules are typically low powered and communicate wirelessly with one gateway in a star fashion. In addition to its communication capabilities, the IQRF TR module includes an AES 128-bit encryption co-processor, providing the means for IQRF to implement its security mechanisms. Figure 5 shows a simplified IQRF TR architecture. The second level of the IQRF system architecture is the IQRF gateway software, called IQRF GW-Daemon. IQRF gateway is a powerful open source software package allowing to easily create a link to the internet and cloud connectivity. Typically, it runs on an embedded Linux-based single-board computer such as RaspberryPi. On the single-board computer, the coordinator TR is mounted as shown in Figure 2. The GW-Daemon gateway utilizes multiple communication channels, i.e., UDP, MQTT, and WebSocket, and can be managed through a web interface called Webapp or through terminal commands. Finally, the last level of IQRF system architecture is where the cloud system resides. In this article, the type of the cloud is not defined. However, as in most IoT networks, it represents the edge of the system that would store and parse the data sent from the nodes [8].

### 4.2. Identify Failure Modes

Considering the aforementioned IQRF architecture and the focus of the article; in this study, four main failure modes are identified which are tied to the distinct architectural components, namely, end nodes, coordinator, gateway, and communication.

1. Compromised End Nodes: Node compromise is one of the most common and detrimental attacks in WSNs. This is due to the pervasive nature of WSNs and the limitation in computational and storage capabilities of the deployed end devices. In fact, the end nodes can be installed in an environment where the attacker can have physical access or eavesdrop the network freely with no restrictions [37]. Hence, any malicious activity that occurs at or targets IQRF end nodes may cause a failure.
2. Compromised Coordinator: IQRF networks require at least one coordinator. On analyzing the network setup shown in Figure 3, it is found that all communications between the end nodes pass through the coordinator node. In addition, this node plays the role of an interface for the end nodes to reach the gateway and then the internet. Therefore, any malicious activity that occurs at or targets the coordinator node can potentially cause a failure [38].
3. Compromised Gateway: IQRF system uses a web interface, Webapp, that is used to access the IQRF GW-Daemon in order to manage the IQRF WSN by configuring nodes and running the joining process procedure. Moreover, the IQRF gateway connects the IQRF WSN to the internet. Hence, any malicious activity that occurs at or targets the IQRF gateway may also cause a failure. For instance, it is possible a hacker may try to steal the information exchanged between the IQRF gateway and the WSN or the information that is transmitted to internet by exploiting the gateway device vulnerabilities [39].
4. Compromised Communication: Any malicious activity that occurs at or targets the communication paths between the different end nodes and the coordinator is another type of failure that needs to be considered. Sending a fake joining request, intercepting a packet, or jamming or replaying an attack can disrupt the communication [38,40].

### 4.3. Identify Existing Controls

In the literature, there are not enough available resources describing the current security controls for IQRF technology in detail except some useful information found in IQRF technical documents [41]. This is due to the fact that IQRF security information has been confidential until now, and that the development of IQRF technology is still an ongoing process. However, in this subsection, the available IQRF technical documents and an investigation of the built set-up shown in Figure 3 are used to identify the current practical controls in place for data protection in an IQRF network.

Basically, in wireless communication, every wireless system is exposed to potential over-the-air (OTA) threats. Hence, since the development of the OS version v4.00, IQRF Alliance has put a special focus primarily concentrated on the security of IQRF networks [8,41]. Starting from this version, IQRF OS implements security based on industrial standards, ensuring authorized access to OTA flowing data. IQRF OS adopts the industrial standard AES-128 for wireless communications encryption. Thus, besides hiding sensitive information, data encryption increases consistent protection and prevents packet forging.

In an IQRF network, there are mainly three different levels of protection, all are based on AES-128 [42]:

1. Access Encryption: this is an independent encryption that is always applied when joining the network.
2. Network Encryption: all networking communications are encrypted.
3. User Encryption: payload data packets can optionally be encrypted by a user specific key in the user application code to hide its content.

These three levels of protection are systematically and respectively involved in three protection procedures that IQRF technology uses to protect its network, such as joining an IQRF network by an unjoined node, circulating data protection over an operating IQRF network, and the provided optional user encryption. In the same order, the three protection procedures are described in detail in the following subsections.

#### 4.3.1. Joining a Node to an IQRF Network

Joining a wireless network is an important procedure from a cyber security point of view. This procedure decides whether a node is allowed to join a given network or not. Therefore, in an IQRF network, only authorized devices and users with a valid access key are allowed to join the network. Additionally, compromising keys is very frequent source of security threat. Therefore, the IQRF OS takes charge of the generation and manipulation of access keys. For instance, the access key for a node to join a network is unknown during physical manipulation since this key is generated from a 16 Byte (B) length password, called an access password, that is specified by the user or the network developer during node configuration when the network is built for the first time. Thus, deep inside the OS, the access key is generated using the access password, and it is modified with an embedded hash function. Therefore, there is no simple or direct relationship between the access password and the generated access key. This increases the access security level within an IQRF network. On the other hand, the relationship between passwords and keys is never the same in different networks. Hence, breaking a key in one network has no impact on other networks. Additionally, the keys are generated dynamically, and change over time, which makes the IQRF network more immune to several types of specific attack. In the end, the user has to take care of passwords and never handles keys, since the key management and distribution are entirely handled by the OS.

**Figure 6:**
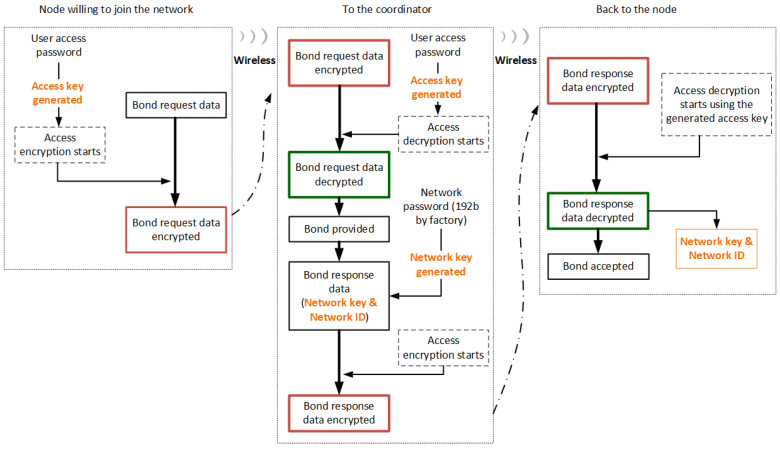
Protection during joining the network process (simplified) https://www.iqrf.org/technology/security, accessed on 20 October 2022.

During the joining process, all sensitive data, such as networking passwords described in Section 4.3.2, the network identification (NID), and the node address of the node willing to join the network, are transferred encrypted. At this level, AES-128 with a 16 B long key and the standard electronic codebook (ECB) mode of operation are used for encryption [41]. The joining process of a node to an IQRF network is simplified in Figure 6.

#### 4.3.2. Protection of Data Sent over IQRF Network

**Figure 7:**
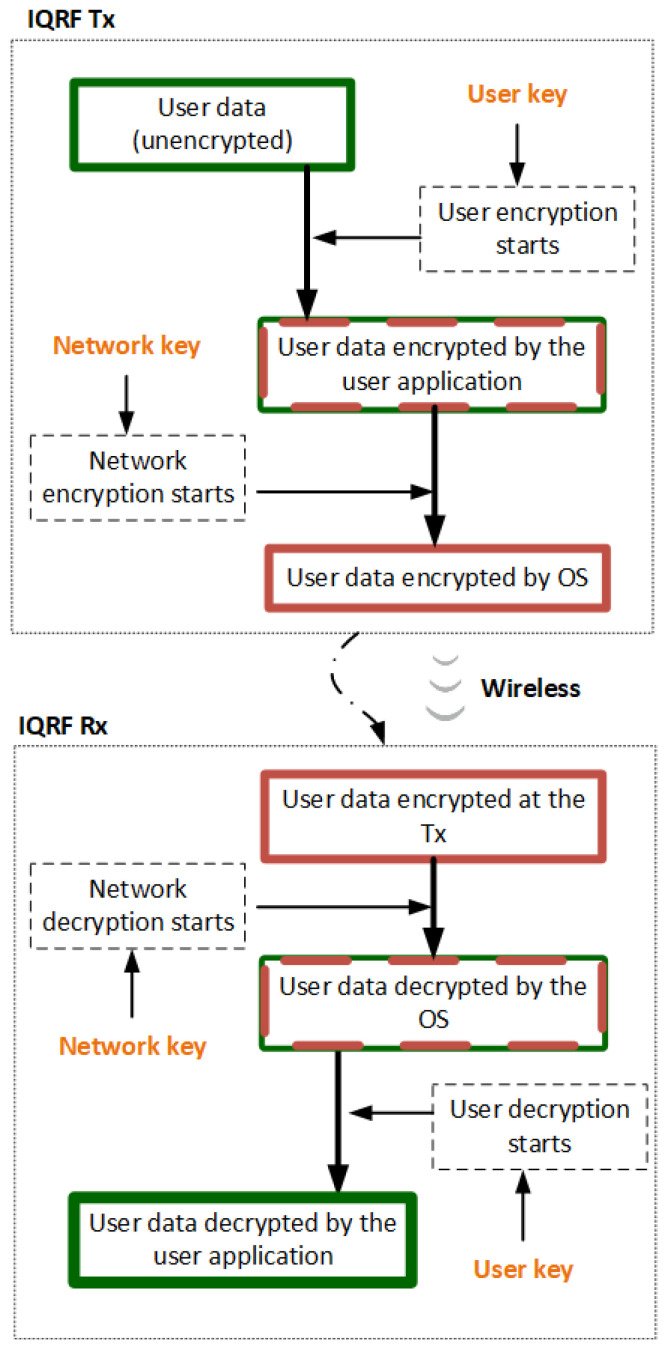
Protection during networking communication (simplified) https://www.iqrf.org/technology/security, accessed on 20 October 2022..

Generally, an IoT network architecture is expected to be faced with an often changing threat environment, with attackers always trying to find and exploit its vulnerabilities. Therefore, the protection of standard network communication becomes important, since it continues during the whole time of operation. For this reason, all packets circulating in an IQRF network are encrypted automatically by the OS, and only nodes with a valid networking key are allowed to communicate and to process the data. Similar to access key generation, a 16 B networking key is generated by the OS using a 24 B password called the networking password. The networking password is a unique password generated individually and randomly with high entropy and installed at each IQRF TR (node) during the manufacturing process. It is worth mentioning that the networking key is only generated at the coordinator node using its 24 B networking password and is transferred encrypted to the nodes willing to join the network during the joining process, as shown in Figure 6. This means that the networking passwords stored in other nodes rather than the coordinator node are not used, unless one of these nodes is configured as a coordinator in another scenario. This process is abstract to the user and is completely controlled by the OS [41]. Figure 7 shows the protection of data sent over an IQRF network.

#### 4.3.3. Optional User Encryption

In addition to data encryption, by using an access key and an networking key in an IQRF network, the user can also contribute to increasing the data privacy and security by adding another encryption shield in order to hide the user’s payload for both networking (using IQMESH) and non-networking (peer-to-peer) schemes [6,43]. Accordingly, user encryption is fully handled and controlled by the user in the user application code. Hence, a 16 B user key needs to be specified at the transceiver (node) configuration phase or in the user application code by first placing the desired 16 B user key at a buffer called bufferINFO, and then setting the user key using the embedded OS function named setUserKey(). Once the key is set, the OS encryption function, named encryptBufferRF(blocks), needs to be called. On the other hand, at the receiving node, a decryption OS function, named decryptBufferRF(blocks), must to be called for decryption. This function replaces the ciphertext in the bufferRF by the plaintext decrypted by the current user key. The ciphertext must be a multiple of 16 B (16 B, 32 B, 48 B, or 64 B). In addition, the same key must be used for encryption and decryption. Figure 7 also shows the user data encryption.

### 4.4. Identify Effects of Failure Modes

The effects of failure modes are sensitive to the use case in which the IQRF technology is hosted. The effects of failure modes triggered due to cyber attacks can be categorized according to the confidentiality, integrity, and availability (CIA) triad for use cases relevant to information technology (IT) [44]. However, if the use case is categorized as a cyber physical system (CPS), other effects should be considered, such as safety and operational impacts [33]. The analysis conducted in this study considers a generic application and discusses the expected consequences in general. Considering the identified failure modes, the possible consequences can be summarized as follows:

- Revealing Sensitive Information: if IQRF technology is utilized for communicating sensitive information, then revealing such information and violating their confidentiality could be a possible objective of cyber attacks.
- Operational Impact: Attackers may target the service provided through the IQRF technology and aim to disrupt it in a manner similar to denial of service. This can target data availability and/or integrity, which may result in a financial impact as well as a possible safety impact if the IQRF technology is involved in safety-critical use case.

Notably, these effects are not comprehensive; other unforeseen consequences might surface based on the utilization of the IQRF technology. Therefore, the FMEA process should be iterated upon further definition of the use case.

### 4.5. Identify Failure Causes

Regarding the security mechanisms implemented by IQRF technology, it should be possible to use IQRF solutions securely to protect against attacks such as man in the middle threading the confidentiality and integrity of circulating packets [45]. However, other areas are left to the developers, which may lead to security vulnerabilities that could be specific to IQRF use cases. The following sections highlight the responsibilities of the developer and also describe other types of attacks that could be performed and might cause failures if such vulnerabilities take place.

#### 4.5.1. Weaknesses in Key Generation and Sharing

As mentioned earlier, the access key and the networking key are generated by the OS using the access password and the networking password, respectively. Moreover, both keys are not directly derived from the passwords, but also modified by embedded hash functions. This results in that there is no direct relationship between the passwords and keys. Besides, for a given IQRF network, the used networking password is the one of the coordinator nodes, as shown in Figure 6. IQRF specifications require that the networking password is passed encrypted to the new device willing to join an IQRF network during the joining process using only the access encryption. A weak access password will lead to a weak access key generation. Hence, finding the the access key is possible using brute force techniques. Consequently, this will lead to finding the networking key. Intercepting the networking key may expose the system to serious threats, since it is used during the network operation. Therefore, an attacker with the networking key might be able to intercept the traffic from any node in the network [14,46,47].

#### 4.5.2. Weaknesses in Key Management

In an IQRF network, the coordinator node is ultimately responsible for the management of the networking key. The process of generating and storing keys could introduce vulnerabilities that undermine the security offered by IQRF. Since the symmetric key AES-128 algorithm is used to encrypt data, at least two keys must be set into two different nodes, e.g., the coordinator and the end node in cases where the network is set using two nodes only. Once keys are generated, both end nodes and the coordinator should be storing the generated keys in a specific memory space. Therefore, it is likely given the range of physical attacks available that an attacker could get the access key and the networking key from the node by accessing the memory where the keys are stored. Consequently, with a hacked key, the attacker would be able to reproduce or hack the encrypted data.

#### 4.5.3. Failure during Joining the Network

In a given IQRF network, end nodes are paired with the coordinator node. This process is a basic step of the network installation. Moreover, the joining operation is accomplished over the air by exchanging sensitive information such as the networking key and NID between the coordinator node and the end node willing to join the network. Sensitive information is exchanged encrypted by access encryption. However, if the access key is revealed due to a weak access password for instance, it is possible for an attacker to find the networking key and to act as one of the end nodes in the network [48].

#### 4.5.4. Physical Attack

**Figure 8:**
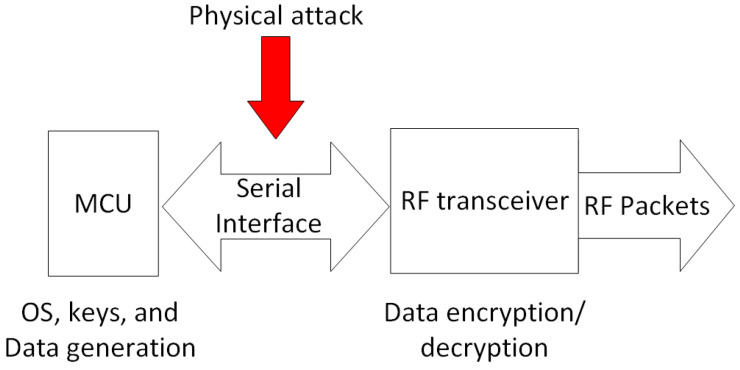
Physical attack of a typical and simplified IQRF node setup.

In many cases, IoT devices are used in remote locations. In fact, those networks are exposed to physical attacks ranging from tempering through to theft. Therefore, an IQRF node can be compromised in a number of ways. For instance, from an IQRF transceiver’s basic architecture, shown in Figure 8, and the explored technical documents of the used components and circuits, it is observed that the MCU communicates with the RF circuit using serial communication interfaces such as SPI or UART. All data, such as the generated networking and access keys by the OS and the NID in the case of the coordinator node, are exchanged via the serial interface un-encrypted to the RF circuit. This is due to the fact that all generated data from the MCU are encrypted/decrypted at the RF circuit where the AES 128-bit encryption co-processor is located. Thereupon, an attacker with physical access to one of the network end nodes (IQRF TRs) could in theory packet sniff using signals recorded on the serial interface of the IQRF transceiver. Therefore, the access and the networking keys and all the sensitive information could be recorded.

#### 4.5.5. Message Integrity Code (MIC) Failure

Usually, coordinator nodes and end nodes in a network are enabled with mechanisms for checking the received messages integrity as well as maintaining counters for messages. A message integrity code (MIC) provides a strong assurance of authenticity. However, the use of an MIC mechanism is not mentioned in the IQRF specifications. The IQRF rather uses a mechanism called the packets consistency check (PCC) to eliminate transmission and environment errors. The PCC is also mentioned to be used to protect IQRF networks against malicious misuse in some cases. The mechanism assumes that received packets are checked against bit error failures using several block checksums based on the IBM CRC-16 standard. Basically, the cyclic redundancy check (CRC) is designed to detect only accidental errors in the received data. On the other hand, the CRC can be reproduced by an intruder [49]. However, the MIC detects intentional and unauthorized modifications of the data as well as accidental errors. Hence, in the case of IQRF, the lack of an MIC mechanism will enable reply attacks, whereby messages exchanged between nodes can be recorded and played back in the network.

#### 4.5.6. Gateway Compromise

The IQRF gateway is connected to internet using an IP connection and acts as a bridge for IQRF nodes to the internet. For other attacker, a gateway may be considered as an advantage to route gateway traffic through a private network. In this case, several attacks are relevant, such as gaining access to the IQRF gateway through compromised credentials on Webapp. Our analysis demonstrated that HTTP is enabled and active, as is the case for HTTPS. When HTTP is used to access the gateway, attackers in the same network are able to reveal the credentials through sniffing using a tool such as Wireshark. To mitigate this threat, accessing the gateway through HTTPS only is advised. Additionally, there is no enforced password policy at the gateway when creating accounts on Webapp. This allows for weak credentials which can be brute forced. Therefore, utilizing strong credentials is advised. Moreover, physical attacks are of additional concern and thus physical security need to be considered. In the case of a compromised gateway, the entire IQRF network becomes vulnerable. Attackers could impact the network structure, disrupt or alter its behavior, and violate privacy and information confidentiality in the case of IQRF network implementation in a system that deals with sensitive information. Nevertheless, the security of the IQRF gateway is not within the main scope of this article and is not pursued any further.

### 4.6. Evaluate Risks

Due to the limited documentation about IQRF security and the limited research conducted regarding this technology, the work presented in this study is limited to a qualitative evaluation of risks by discussing the impact of the defined failure modes, highlighting especially the ones that would have the biggest impact, and also by discussing the occurrence likelihood of its failure causes (i.e., attacks) that are more feasible in the built IQRF network of Figure 3.

The most important security issues considered in this study are cyber attack exposure and high risk vulnerabilities in critical devices such as the coordinator and the gateway. Moreover, disrupting the communication such as causing interference in the wireless communication (i.e., jamming attack) is also a critical issue. For gateway compromising, as discussed in Section 4.2, failure modes have the ability to configure the IQRF network and control the network joining procedures and some other system configurations. An attacker with access to a compromised gateway has the ability to cause serious damage to the network functionality, which would be reflected in various ways depending on the use case. On the other hand, compromising the coordinator node failure mode can also cause serious damage to the whole IQRF network in cases where only one coordinator is managing the network, or to a portion of IQRF network in cases where there are multiple coordinator nodes. The effect of compromising the wireless communication that links the end nodes together would differ based on the targeted communication path. Therefore, if an attacker is capable of compromising the communication going from coordinator to the end nodes (i.e., data alteration), the impact would be relatively high, as several end nodes will receive altered packets. One other mentioned failure mode in Section 4.2, and one that also needs to be evaluated, is the end node compromise. Compromising one end node will theoretically have no impact on the other neighboring nodes, including the coordinator. However, if the end node is critical in the network, for instance, nodes playing the role of routers that the IQMESH routing protocol uses to route information in an IQRF network, the case might be different. A well-known attack in this case would be a sinkhole attack. Although the impact is less severe compared to the other three failure modes mentioned above, this attack can still disrupt a part of the network.

From the failure cause occurrence likelihood perspective, compromising keys represents one of the highest risks for any key-encrypted wireless network. This is a very important issue as far as network security is concerned. If an attacker gets hold of a key, they will be able to act at leisure within the network. For this reason, key generation, sharing, and management are three important mechanisms to point out in case of IQRF. Access key generation in IQRF depends on the access password used by the network developer (maker). Therefore, a weak access password choice makes an IQRF network vulnerable to the password brute force type of cyber attack. With a revealed access password, this will expose the networking key generated by the coordinator and shared with the node willing to join the network. Another failure cause that is also concerning is the storage of the keys. By knowing an IQRF transceiver’s main components, including the ones where the different keys could be stored, motivated attackers might gain physical access to an IQRF network through the acquisition of a node and by gaining control over the serial communication interface between the MCU and the storage or the RF units, as shown in Figure 8. Hence, this represents another point of failure for an entire system. Furthermore, replay attacks are relatively easy to implement if the MIC mechanism is not properly implemented, such as in the case of an IQRF where a PCC mechanism (based on IBM CRC-16) is used instead. Attackers may passively collect the communicated packets within the network and replay them at later stage to cause undesired outcomes. This assumption is valid if it is assumed that the attackers have successfully gained access to the network. In the end, countering replay attacks, physical attacks, and compromised gateways and coordinators should receive the highest priority.

### 4.7. Identify Actions

From the vulnerabilities mentioned previously, it can be summarized that the weaknesses in both design and implementation may expose an IQRF system to several attacks. However, it is possible to build an IQRF solution that can prevent, detect, and respond to cyber attacks.

Prevention of attacks starts at the joining process, since this process is accomplished over the air by exchanging some system RF packets. Therefore, in order to avoid the entry of an unwanted entity, the system encrypts sensitive information, such as the NID and networking key, and shares them with the new device willing to join the network. Still, the access encryption is a strong mechanism that is implemented in IQRF networks based on AES. However, it is the responsibility of the network developer to choose a strong access password. Hence, considering good security practices, such as using random keys, helps to prevent attacks targeting the keys. Additionally, on the gateway side, when using the configuration interface (Webapp), good practices in setting the login password are also required. On the other side, physical attacks can be prevented using physical security. This is a practice of vital importance to prevent the system from unauthorized persons gaining physical access to the network and causing harm. Therefore, physical security or intrusion detection mechanisms protecting the hardware, such as the gateway, from being tampered with need to be considered. In addition, prevention of the attacks mentioned in this study is required. However, this requires the inspection and reporting of the identified attacks to the developers or network maintainers. An example of an attack seeking to modify encrypted packets could still be identified by the PCC mechanism as an unusual incident such as that caused by radio frequency interference. Nevertheless, an MIC mechanism should be implemented in IQRF future OS versions to make the system more immune to message integrity attacks.

## 5. Discussion

An IQRF network can be built in different ways depending on its application. The setup used in this study is a general case scenario of an IQRF network, as described in Section 3.2. Furthermore, challenges are encountered in finding resources based on research and technical data describing IQRF security. Therefore, the scope of this study is limited to a qualitative analysis using the FMEA approach. As defined in the IEC 60812:2018 standard, FMEA provides qualitative information originally lacking in severity ratings, occurrence, detection rankings, and the criticality matrix compared to its complementary approach FMECA, which provides qualitative and quantitative information, allowing users to measure the criticality level of failure modes and rank them in order of importance. Thus, a criticality analysis of FMECA is left to be performed as an extension of the work in this article.

The findings of this study indicate that the highest risk corresponds to four failure modes that are related to IQRF system architecture, including compromised end nodes, a compromised coordinator, a compromised gateway, and a compromised communication between nodes at the WSN level. These failure modes, in some scenarios, can expose sensitive information within the IQRF network or cause other catastrophic situations if the network is deployed in critical infrastructures. On the other hand, failure causes in some cases can be the source of the failure modes discussed earlier and may also be the source of other failure modes that are not explored in this article. However, in this study, human-related failure causes, such as those related to good practices in choosing the access password, setting up a web interface password for the gateway, and physical threats, or system-related failure causes, such as those involved in network key generation, sharing, and management (as shown in Figure 6), are described in detail and need to be considered as the main focus while designing an IQRF network. In addition, the lack of an MIC mechanism for packet consistency checks in an IQRF network, which needs to be considered as well, can also be a source of additional failure.

Other findings of this study are the qualitative risk evaluation of the parts of the system that may have the largest threat impact on the IQRF network and the suggestion of actions that can help improve future versions of the IQRF OS. Within the IQRF WSN, the coordinator is the critical device as all exchanges are passed through this node, except for those relayed through the end nodes if the end node is used as a router in some scenarios that require multi-hop communication. Moreover, the IQRF gateway and the communication link (channel) between nodes are also critical parts of the system. Hence, any malicious activity that targets one of these parts can cause serious damages to the entire network. On the other hand, compromising the end node will theoretically have no effect on the network and other neighboring nodes. However, if the end node is used as a routing node, it may cause damage to a part of the network. Finally, in addition to good practices and physical security proposed in Section 4.7, the integrity of the circulating information is still important. The CRC-32-based PCC mechanism implemented by IQRF in its networks could be a disadvantage in the event of a true replay attack. This is because the PCC may detect these attacks as an accidental RF packet error. Therefore, it is recommended to implement the MIC mechanism in future versions of IQRF OSs.

## 6. Conclusions

IQRF technology provides secure solutions that could protect IQRF-based WSNs. However, it should be noted that using IQRF solutions does not guarantee optimum security. Instead, building an IQRF network should always be accompanied with a consideration of the potential attacks that could be undertaken. In this article, we aimed to perform a risk analysis for an IQRF network (system). To this end, an FMEA approach is employed. Thus, security risks in IQRF technology are identified and further actions that can be used to protect IQRF networks from cyber attacks are proposed. Moreover, in order to develop a secure IQRF network, detailed information on the failure modes and causes, along with the security features, of the IQRF is provided. For this reason, in this study, reaching a secure IQRF solution by considering cyber security at the main stages of the system, including the WSN level and both the gateway and the cloud (server) level, is not addressed.

On the other hand, while risk analysis is crucial for successfully identifying the failures, causes, and effects, a risk assessment strategy is also required to be developed. Risk assessment is an important step to determine the action plan to improve the cybersecurity of a system. In this context, there are various ways to assess the risk of the failures identified during the FMEA process. One way is to use the RPN, which provides a numerical result that offers better quantification of the risk. However, due to the limited documentation and research literature on IQRF security, the scope of this work is restricted to conducting a risk analysis only. For this reason, the study presented in this article can be considered as an initial study of IQRF cybersecurity. In the near future, the presented work is planned to be extended by performing a risk assessment for IQRF networks.

## Figures and Tables

**Table 1:** Summary of relevant researches on the application of FMEA to IoT-based systems.

Ref.	Application	Aim	Approach
[29]	Honeypot systems in IoT	Identifying and mitigating the challenges in the deployment of honeypots for IoT	FMEA
[30]	Smart city	Assessing the information security risk of smart city	FMEA based on the fuzzy set theory and the gray relational theory
[31]	Smart buildings	Resilience assessment of two case studies on smart buildings	Coupled FRAM–FMEA
Proposed work	IQRF networks	Conducting security risk analysis to protect IQRF networks from cyber attacks	FMEA

## Data Availability

The data presented in this study are available on request from the corresponding author.
